# Comparison of thermal manikins to human thermoregulatory responses

**DOI:** 10.1186/2046-7648-4-S1-A16

**Published:** 2015-09-14

**Authors:** Jonathan Power, Andrew Baker, António Simões Ré

**Affiliations:** 1 National Research Council of Canada, St. John's, NL, Canada

## Introduction

Immersion suits are lifesaving appliances (LSA) designed to protect the wearer if they become accidently immersed in cold water by reducing the cold shock response and delaying the onset of hypothermia. Immersion suits are certified to both national and international standards; some of which require the thermal protective properties to be tested using humans or thermal manikins. The ethical nature of testing with humans has been questioned [1] due to the physically grueling nature of these tests, thus testing with manikins may be preferential. However, previous work has shown that discrepancies exist between thermal manikins and humans that could result in immersion suit selection that would benefit the former more than the latter who would ultimately use it [2]. This study investigated the thermoregulatory responses of humans and compared them to a thermal manikin while wearing immersion ensembles with insulation distributed in various configurations hypothesized to be beneficial to humans and manikins.

## Methods

Eleven healthy males performed three, 3-hour immersion in 5 °C stirred water while wearing various immersion ensembles. The immersion ensembles consisted of standardized underclothing, an outer waterproof shell, and a custom-made closed cell neoprene inner liner with insulation distributed in three configurations: "Control" - insulation distributed evenly around the limbs and torso; "Human Beneficial" (HB) - insulation concentrated around the torso compared to the limbs; and "Manikin Beneficial" (MB) - insulation concentrated around the limbs compared to the torso. Mean skin temperature (T_SK_), mean skin heat loss (MSHL), gastro-intestinal temperature (T_GI_), and oxygen consumption (VO_2_) were measured throughout the immersions. A 23 zone thermal manikin (NEMO) was also immersed with the same three ensembles.

## Results

For the humans, across all ensembles, there were no significant differences in the mean (SD) change in T_SK _(Control: -4.5[0.6] °C; HB: -4.5[1.0] °C; MB: -4.6[0.9] °C), MSHL at the end of the immersions (Control: 95.5[10.2] W·m^-2^; HB: 101.9[8.2] W·m^-2^; MB: 102.0[8.8] W·m^-2^), change in T_GI _(Control: -0.1[0.4] °C; HB: -0.2[0.4] °C; MB: -0.3[0.3] °C), and VO_2 _at the end of the immersion (Control: 515.7[79.5] mL·min^-1^; HB: 538.9[77.3] mL·min^-1^; MB: 565.3[101.2] mL·min^-1^). There were no significant differences in the clo value of the ensembles as measured by the humans (Control: 1.39[0.16] clo; HB: 1.28[0.13] clo; MB: 1.28[0.15] clo); and NEMO (Control: 1.09 clo; HB: 1.06 clo; MB: 1.09 clo).

## Discussion

Our findings do not agree with the previous work [2] undertaken with lower levels of overall insulation in that we did not find any significant differences in change in deep body temperature (T_GI_) or VO_2 _when insulation was reduced over the torso compared to the limbs. As well, when insulation was reduced over the limbs compared to the torso, NEMO did not measure a large (>10%) drop in clo. Initial results suggest that all three ensembles caused the same level of thermal stress in the humans, even though insulation was concentrated around the torso in HB, and reduced around it in MB.

## Conclusion

Higher overall insulation values may negate the differences in thermoregulatory and thermal responses of humans and manikins evoked by differences in regional insulation.
